# Reduction in Respiratory Syncytial Virus (RSV)‐Related Outpatient and Inpatient Cases Among Infants During the Initial 2024/2025 Season Following Nirsevimab Recommendation in Germany

**DOI:** 10.1111/irv.70274

**Published:** 2026-06-17

**Authors:** Wei Cai, Isabelle Maronde, Sophie Köndgen, Barbara Biere, Silke Buda, Brunhilde Schweiger, Doreen Staat, Walter Haas, Ekkehard Schuler, Virologic SARI Surveillance Study Group, Thorsten Wolff, Ralf Dürrwald, Djin‐Ye Oh, Kristin Tolksdorf, Janine Reiche

**Affiliations:** ^1^ Unit 36, Respiratory Infections, Department of Infectious Disease Epidemiology Robert Koch Institute Berlin Germany; ^2^ Unit 17, Influenza and Other Respiratory Viruses, Department of Infectious Diseases, National Influenza Centre Robert Koch Institute Berlin Germany; ^3^ Unit 17, Influenza and Other Respiratory Viruses, Department of Infectious Diseases, Consultant Laboratory for RSV, PIV and HMPV Robert Koch Institute Berlin Germany; ^4^ Department of Infectious Disease Epidemiology Robert Koch Institute Berlin Germany; ^5^ HELIOS KLINIKEN GmbH Berlin Germany

**Keywords:** disease severity, hospitalised, infants, nirsevimab, outpatient, resistant mutations, respiratory syncytial virus, surveillance

## Abstract

After introducing nirsevimab during the 2024/2025 respiratory syncytial virus (RSV) season in Germany, RSV activity in infants under 1 year dropped by 50% in primary care and 50%–70% in secondary care. Inpatients under 6 months showed the greatest reduction. RSV severity decreased with fewer RSV‐associated ICU admissions and shorter hospital stays. RSV sequence analysis identified four known polymorphisms in the nirsevimab binding site among inpatients; none associated with resistance. These findings indicate that nirsevimab effectively reduced medically attended RSV disease in infants.

## 1 Introduction

Respiratory syncytial virus (RSV) is a leading cause of acute lower respiratory tract infections (ALRI) and ALRI‐related hospitalisations in infants, creating a significant global disease burden. RSV has been included in sentinel surveillance systems for acute respiratory infections (ARIs) in primary and secondary healthcare in Germany for many years [1]. To strengthen surveillance, the infection has been notifiable since July 2023. Laboratory‐confirmed acute RSV infections must be reported to health authorities using the German national notification system under the Infection Protection Act.

The European Medicines Agency has recently approved nirsevimab, a long‐acting monoclonal antibody targeting a highly conserved epitope on the RSV fusion (F) protein [2, 3], to prevent RSV‐induced ALRI requiring medical attention in infants. Several European countries introduced passive immunisation with nirsevimab for infants in the 2023/2024 season [4, 5, 6]. In June 2024, the German Standing Committee on Vaccination (STIKO) recommended a single dose of nirsevimab for all infants born during the RSV season (usually between October and March) immediately after birth and for all infants born between April and September in the fall (between September and November). While the immunisation rate for infants born during the 2024/2025 RSV season has not yet been reported, coverage among infants born before the 2024/2025 RSV season (April–September 2024) was 54% [7].

This study aimed to investigate the impact of passive nirsevimab immunisation on RSV‐related diseases in infants under 1 year old, as well as the occurrence of polymorphisms in the nirsevimab binding site of the RSV F protein during the 2024/2025 RSV season in Germany.

### 1.1 Reduced RSV Activity

**FIGURE 1:**
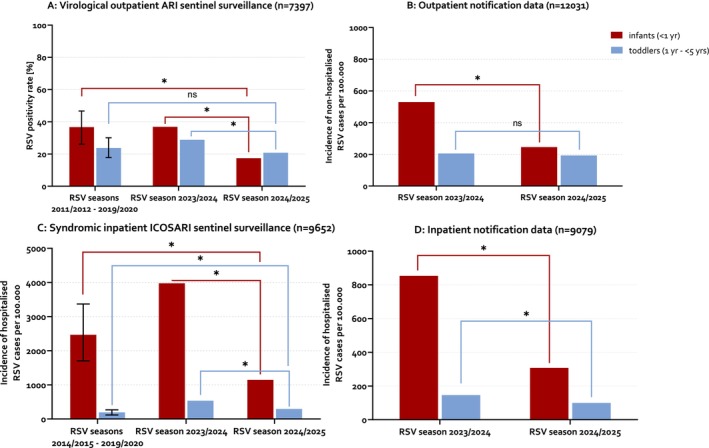
RSV positivity rate and cumulative incidence of RSV cases among outpatient and inpatient children by age group and RSV season, Germany, 2011–2025. (A) The error bar shows the range of the lowest and highest seasonal RSV positivity rate from the 2011/2012 to 2019/2020 RSV season. (B) Outpatient notification data show the cumulative incidence of reported nonhospitalised laboratory‐confirmed medically attended RSV diseases per 100,000 inhabitants. (C) Cumulative incidence of hospitalised RSV cases is given per 100,000 inhabitants. RSV cases are defined as SARI cases with one of the three RSV‐specific ICD‐10 codes (J12.1, J20.5, J21.0) as primary or secondary discharge diagnosis [1]. The error bar shows the range of the lowest and highest seasonal cumulative RSV incidence from the 2014/2015 to 2019/2020 RSV season. (D) Inpatient notification data show the cumulative incidence of reported hospitalised laboratory‐confirmed RSV medically attended diseases per 100,000 inhabitants. Chi‐squared test was used to compare RSV positivity rate and cumulative incidence by age in the 2024/2025 RSV season with that in previous RSV season(s). A *p*‐value of < 0.05 was considered statistically significant (*), a *p*‐value of ≥ 0.05 was considered to indicate no significant difference (ns). ARI, acute respiratory infection; ICOSARI, ICD‐10‐code‐based surveillance for severe ARI; ns, not significant; RSV, respiratory syncytial virus.

Primary healthcare data from our virological surveillance of ARIs [1] and the German national notification system, as well as secondary healthcare data from syndromic ICD‐10‐code‐based surveillance for severe ARI (ICOSARI) [1] and notification system, were analysed for RSV‐infections in infants under 1 year old and in toddlers aged 1 to < 5 years during the 2024/2025 RSV season, compared to the previous 2023/2024 RSV season and pre‐COVID‐19 RSV seasons (Figure 1) (Supporting Information). For clarity, the 2021 and 2022/2023 RSV seasons were not included in the comparison due to their atypical seasonality during the COVID‐19 pandemic [1].

In primary healthcare, both the RSV‐positivity rate (PR) among infants (17%, 29/167) and the cumulative incidence of reported nonhospitalised RSV cases (246/100,000) were approximately 50% lower in the 2024/2025 RSV season compared to the 2023/2024 RSV season (PR: 37%, 94/255; incidence: 530/100,000; risk ratio (RR): 0.46, 95% CI: 0.44–0.49; *p* < 0.001). The RSV‐PR in 2024/2025 was also about 50% lower than in pre‐COVID‐19 RSV seasons (2011/2012 to 2019/2020; 37%, 350/950) (*p* < 0.001) (Figure 1A,B). The RSV PR declined by around 30% in toddlers in the 2024/2025 season compared to the 2023/2024 season. This reduction was smaller than that observed in infants (Figure 1A).

The cumulative incidence of hospitalised RSV cases in infants was approximately 60%–70% lower in secondary healthcare in 2024/2025 (ICOSARI: 1147/100,000; RR: 0.29, 95% CI: 0.26–0.32; notification data: 308/100,000; RR: 0.36, 95% CI: 0.34–0.38; *p* < 0.001) than in 2023/2024 (ICOSARI: 3976/100,000; notification data: 854/100,000). Based on ICOSARI data, the 2024/2025 RSV incidence was approximately 50% lower than the mean seasonal incidence in pre‐COVID‐19 seasons (2014/2015 to 2019/2020; 2468/100,000; RR: 0.46, 95% CI: 0.41–0.52; *p* < 0.001) (Figure 1C,D).

Furthermore, the incidence of hospitalised RSV cases among toddlers was higher in the 2024/2025 season than in pre‐COVID‐19 seasons and declined by approximately 30%–50% compared to the 2023/2024 season. This reduction was smaller than that observed in infants (Figure 1C,D).

### 1.2 Reduction of Severe RSV Disease

In the secondary healthcare (ICOSARI), the proportion of RSV diagnoses among hospitalised severe ARI (SARI) cases was significantly lower in infants in 2024/2025 than in previous RSV seasons investigated (Table 1). The cumulative incidence of RSV cases admitted to intensive care units (ICUs) in infants was 75% lower in 2024/2025 than in 2023/2024 (RR: 0.25, 95% CI: 0.17–0.35; *p* < 0.001) and 32% lower than the mean seasonal incidence in pre‐COVID‐19 seasons (RR: 0.68, 95% CI: 0.45–1.0; *p* = 0.04). The length of hospitalisation was significantly shorter compared to pre‐COVID‐19 seasons, while hospitalised RSV cases were somewhat older in the 2024/2025 RSV season than in pre‐COVID‐19 seasons (median age of 4 versus 3 months, *p* = 0.0155). The proportion of RSV‐related in‐hospital deaths remained very low across all RSV seasons and showed no significant variation.

**TABLE 1:** Characteristics of hospitalised RSV cases aged < 1 year by season, ICOSARI surveillance, Germany, 2014–2025 (*n* = 15,834).

Variable	Pre‐COVID‐19 RSV seasons 2014/2015 to 2019/2020	RSV season 2023/2024	RSV season 2024/2025	Comparison pre‐COVID‐19 and 2024/2025 season *p*‐value	Comparison 2023/2024 and 2024/2025 season *p*‐value
Number of hospitalised cases with RSV^a^ diagnosis	838^b^ (male 57%)	1175 (male 59%)	322 (male 56%)	NA	NA
Median age of hospitalised RSV cases	3 months (IQR: 1–6)	3 months (IQR: 1–6)	4 months (IQR: 1–7)	0.0155^g^	0.0521
Cumulative RSV hospitalisation incidence per 100,000	2468^c^	3976	1146	< 0.001^d^ (RR: 0.46, 95% CI: 0.41–0.52)	< 0.001^d^ (RR: 0.29, 95% CI: 0.26–0.32)
Proportion of RSV diagnoses among SARI^e^ cases	40% (5028/12647) (95% CI: 39–41)	55% (1175/2135) (95% CI: 53–57)	31% (322/1052) (95% CI: 28–34)	< 0.001^d^	< 0.001^d^
Cumulative ICU‐admitted RSV incidence per 100,000	182^f^	495	124	0.04^d^ (RR: 0.68, 95% CI: 0.45–1.0)	<0.001^d^ (RR: 0.25, 95% CI: 0.17–0.35)
Proportion of deaths among hospitalised RSV cases	0.1% (3/5028) (95% CI: 0.0–0.2)	0.1% (1/1175) (95% CI: 0.0–0.5)	0% (0/322)	0.661^d^	0.601^d^
Median length of hospital stay among hospitalised RSV cases	4 days (IQR: 3–6)	4 days (IQR: 2–6)	3 days (IQR: 2–5)	<0.001^g^	0.252^g^

^a^ A RSV case is defined as a SARI case with one of the three RSV‐specific ICD‐10 codes (J12.1, J20.5, J21.0) as primary or secondary discharge diagnosis [1].

^b^ Mean number of seasonal hospitalised cases with RSV diagnosis for RSV seasons 2014/2015 to 2019/2020.

^c^ Mean cumulative RSV hospitalisation incidence for RSV seasons 2014/2015 to 2019/2020.

^d^ Chi‐squared test. A *p*‐value of < 0.05 was considered statistically significant.

^e^ A SARI case is defined as a hospitalised case with one of the ALRI ICD‐10 code diagnoses (J09–J22) as primary or secondary discharge diagnosis [1].

^f^ Mean cumulative ICU‐admitted RSV incidence for RSV seasons 2014/2015 to 2019/2020.

^g^ Mann–Whitney U test. A *p*‐value of < 0.05 was considered statistically significant.

ICU, intensive care unit; IQR, interquartile range; NA, not applicable; RR, risk ratio; RSV, respiratory syncytial virus; SARI, severe acute respiratory infection; 95% CI, 95% confidence interval.

### 1.3 RSV Cases Declined Most in Infants < 6 Months

**FIGURE 2:**
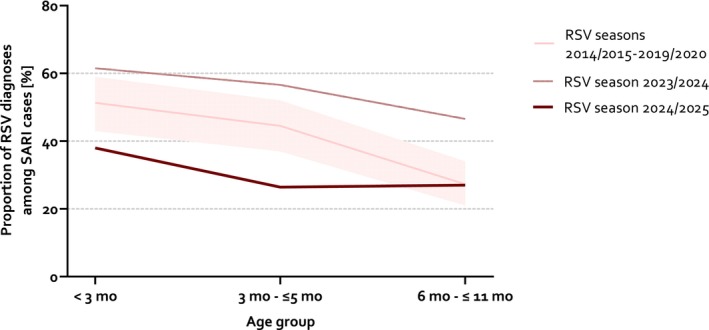
Proportion of RSV diagnoses among SARI cases by age, ICOSARI surveillance, Germany, 2014–2025 (*n* = 15,834). An RSV case is defined as a SARI case with one of the three RSV‐specific ICD‐10 codes (J12.1, J20.5, J21.0) as primary or secondary discharge diagnosis. A SARI case is defined as a hospitalised case with one of the ALRI ICD‐10 code diagnoses (J09–J22) as primary or secondary discharge diagnosis [1]. The red shadow shows the range of the lowest and highest seasonal proportions of RSV diagnoses among SARI cases by age group from the 2014/2015 to 2019/2020 RSV season. RSV, respiratory syncytial virus; SARI, severe acute respiratory infection.

In the secondary healthcare (ICOSARI), the proportion of RSV diagnoses among SARI cases in infants aged < 3 months (38%, 135/355) and 3–5 months (26%, 61/231) was significantly lower in the 2024/2025 season than in pre‐COVID‐19 seasons (< 3 months: 51%, 2164/4216; 3–5 months: 44%, 1463/3290) and in the 2023/2024 season (< 3 months: 61%, 509/828; 3–5 months: 57%, 321/567; *p* < 0.001) (Figure 2). Among infants aged 6–11 months, the proportion of RSV diagnoses in 2024/2025 (27%, 126/466) was also lower than in 2023/2024 (47%, 345/740; *p* < 0.001) but not significantly different from pre‐COVID‐19 seasons (27%, 1401/5141; *p* = 0.921).

### 1.4 Mutations Identified in Nirsevimab Binding Site

As part of the virological SARI surveillance, the introduction of nirsevimab prophylaxis in Germany was also monitored at the molecular level. In 2024/2025, this surveillance identified 204 SARI cases under 1 year old; 42 were RSV positive.

RSV was sequenced in 25 cases using NGS [8], and RSV F protein sequences were analysed for polymorphisms using Geneious (version 2025.0.2), with particular focus on the nirsevimab binding site [2]. In one of the 13 RSV‐A sequences, the F:K65R polymorphism was detected. All 12 RSV‐B viruses shared the F:I206M/Q209R/S211N combination of polymorphisms. One of these viruses was identified in a breakthrough infection in a preterm infant who had received nirsevimab 7 days prior to symptom onset.

## 2 Discussion

This study provides valuable insights into RSV activity and the occurrence of polymorphisms in RSV strains in Germany during the 2024/2025 season, after nirsevimab was introduced. Similar to other countries, RSV activity among infants under 1 year old decreased by 50%–70% in secondary healthcare in Germany [4, 5, 9, 10]. Our study also revealed a notable decrease (by 50%) in RSV activity in this age group within the primary healthcare sector, similar to the reduction of medically attended RSV‐LRTI reported for a primary care network in Spain [11]. Furthermore, the RSV activity among toddlers in the 2024/2025 season declined by 30%–50% compared to the previous 2023/2024 season, potentially due to the indirect effects of infant immunisation on herd protection or household transmission [12].

As described for Luxembourg [5], RSV‐associated ICU admissions and the length of hospital stays were lower among German infants in 2024/2025. Furthermore, the proportion of RSV cases among SARI cases decreased most among infants aged < 3 months and 3–5 months in Germany, which is consistent with findings in other countries [5, 10, 13]. Notably, hospitalised RSV cases were slightly older in 2024/2025 than in the pre‐COVID‐19 seasons. It can be assumed that the shift towards older infants resulted in less severe disease and fewer ICU admissions.

The widespread use of nirsevimab has raised concerns about potential emergence of resistance mutations. In this study, we identified four polymorphisms within the nirsevimab binding site (RSV‐A, F:K65R; RSV‐B, F:I206M/Q209R/S211N), all of which have been previously reported [14, 15]. The F:K65R polymorphism was identified in 1% (275/29,429) of RSV‐A sequences reported globally, and the combined F:I206M/Q209R/S211N polymorphisms were detected in 53% (14,126/26,552) of RSV‐B sequences reported globally during the period from October 2021 to October 2025 (https://gisaid.org/). These polymorphisms do not diminish neutralisation by nirsevimab [14, 15, 16]. Therefore, presence of F:I206M/Q209R/S211N in the RSV‐B strain detected in the breakthrough case is likely coincidental and did not facilitate the breakthrough infection.

We conducted an ecological study in which we analysed representative, population‐based health data from both primary and secondary healthcare settings at a group level. However, the analysis does not account for nirsevimab immunisation status among individual infants. Nevertheless, the nirsevimab immunisation rate was reasonable in Germany; thus, we can assume that the majority of infants in our study received nirsevimab during the 2024/2025 season. Besides, this study does not consider potential indirect competitive population effects related to high influenza activity in 2024/2025 or the impact of enhanced RSV activity in 2023/2024, which may have boosted parental and sibling immunity and thereby reduced infant infection rates in 2024/2025. Nevertheless, consistent with data from Saxony, Germany [17], a significant reduction in RSV activity was observed in infants, particularly when compared to pre‐pandemic seasons. Furthermore, although the inclusion criteria and coding practices for discharge diagnoses remained essentially unchanged throughout the analysed period in both ARI and ICOSARI sentinel surveillance systems [1], the hospitalisation status was unknown for more than a third of the reported RSV cases in the national notification system. This may have resulted in under‐reporting of hospitalised RSV cases within the notification system, thereby reducing the cumulative incidence compared to that reported by ICOSARI.

## 3 Conclusion

Finally, our results indicate that nirsevimab most likely reduces medically attended RSV disease in infants under 1 year of age in primary and secondary healthcare. These results highlight the importance of genomic surveillance in monitoring nirsevimab's impact on RSV disease and the development of resistance.

## Author Contributions

**Virologic Surveillance Study Group SARI:** formal analysis, investigation, writing – review and editing. **Brunhilde Schweiger:** methodology, investigation, project administration, resources, writing – review and editing, conceptualization, formal analysis. **Silke Buda:** conceptualization, funding acquisition, project administration, resources, writing – review and editing. **Isabelle Maronde:** conceptualization, methodology, data curation, investigation, validation, formal analysis, visualization, writing – original draft, writing – review and editing. **Walter Haas:** formal analysis, funding acquisition, resources, writing – review and editing. **Thorsten Wolff:** formal analysis, funding acquisition, resources, writing – review and editing. **Doreen Staat:** data curation, investigation, validation, formal analysis, writing – review and editing. **Wei Cai:** conceptualization, methodology, data curation, formal analysis, investigation, validation, visualization, writing – original draft, writing – review and editing. **Barbara Biere:** conceptualization, methodology, investigation, project administration, resources, writing – review and editing. **Ekkehard Schuler:** methodology, formal analysis, project administration, writing – review and editing. **Ralf Dürrwald:** conceptualization, funding acquisition, project administration, resources, writing – review and editing. **Sophie Köndgen:** methodology, data curation, investigation, validation, formal analysis, writing – review and editing. **Kristin Tolksdorf:** conceptualization, data curation, formal analysis, funding acquisition, project administration, resources, writing – review and editing, methodology, investigation, validation, visualization. **Djin‐Ye Oh:** conceptualization, data curation, formal analysis, funding acquisition, project administration, resources, writing – review and editing. **Janine Reiche:** conceptualization, methodology, investigation, formal analysis, funding acquisition, visualization, project administration, resources, writing – review and editing, supervision, writing – original draft.

## Conflicts of Interest

The authors declare no conflicts of interest.

## Supporting information




**Data S1:** Supporting information.

## Data Availability

The data that support the findings of this study are available from the corresponding author upon reasonable request.
