# Supplementary material for: Reduction in Respiratory Syncytial Virus (RSV)‐Related Outpatient and Inpatient Cases Among Infants During the Initial 2024/2025 Season Following Nirsevimab Recommendation in Germany
Source: Influenza Other Respir Viruses. 2026 Jun 17;20(6):e70274. doi: 10.1111/irv.70274 (PMC13273525; doi:10.1111/irv.70274)
Supplement: Supplementary file 1 — Data S1: Supporting information. [file IRV-20-e70274-s001.docx]

**This supplementary material provides supporting information alongside the article**

**“Reduction in respiratory syncytial virus (RSV)-related outpatient and inpatient cases among infants during the initial 2024/25 season following nirsevimab recommendation in Germany"**

Wei Cai^1^, Isabelle Maronde^2, 3^, Sophie Köndgen^2, 3^, Barbara Biere^2^, Silke Buda^4^, Brunhilde Schweiger^2^, Doreen Staat^1^, Walter Haas^1^, Ekkehard Schuler^5^, Virologic SARI Surveillance Study Group^6^, Thorsten Wolff^2, 3^, Ralf Dürrwald^2, 3^, Djin-Ye Oh^2, 3^, Kristin Tolksdorf^1^, Janine Reiche^2, 3^

shared first authorship: W.C. and I.M.

1. Unit 36, Respiratory Infections, Department of Infectious Disease Epidemiology, Robert Koch Institute, Berlin, Germany

2. Unit 17, Influenza and Other Respiratory Viruses, Department of Infectious Diseases, National Influenza Centre, Robert Koch Institute, Berlin, Germany

3. Unit 17, Influenza and Other Respiratory Viruses, Department of Infectious Diseases, Consultant Laboratory for RSV, PIV and HMPV, Robert Koch Institute, Berlin, Germany

4. Department of Infectious Disease Epidemiology, Robert Koch Institute, Berlin, Germany

5. HELIOS KLINIKEN GmbH, Berlin, Germany

6. Members are listed at the end of the Article

**Supporting Information**

**Data sources**

**Virological outpatient acute respiratory infection (ARI) sentinel surveillance:** Sentinel practitioners voluntarily send weekly nasal and/or throat swabs from symptomatic patients with ARI to the National Influenza Centre at the Robert Koch Institute, Berlin, for laboratory testing for a panel of respiratory viruses, including RSV, on a year-round basis (1)).

**Syndromic inpatient ICD-10 code based hospital surveillance for severe acute respiratory infections (ICOSARI sentinel surveillance):** Data on age, sex and region, admission date, discharge date, length of stay in hospital and in intensive care unit, duration of ventilatory support and outcome of hospitalised patients with any of the respiratory ICD-10 codes (chapter X in (2)): J00-J99) as primary or secondary discharge diagnosis were sent weekly to RKI on a year-round voluntary basis (1). In addition, a RSV case is defined as a SARI case with one of the three RSV-specific ICD-10 codes (J12.1, J20.5, J21.0) as primary or secondary discharge diagnosis. Further, a SARI case is defined as a hospitalised case with one of the ALRI ICD-10 code diagnoses (J09–J22) as primary or secondary discharge diagnosis.

**German national notification system:** In accordance with the German Infection Protection Act, laboratories and medical doctors have been required to notify the respective local health authority of any detection of acute RSV infection since July 2023 (3). This also applies to rapid tests performed in medical practices. These reports must be submitted to the local health department within 24 hours of detection. The local health authority identifies further patient information, such as hospitalisation status, and then transmits the data to the relevant state health authority. From there, the data are sent to the Robert Koch Institute.

**Data analyses**

Using virological outpatient ARI sentinel surveillance, the onset and offset of each analysed RSV season were defined as the first of two consecutive weeks in which the lower limit of the 95% confidence interval of the RSV positivity rate (PR) in children under five years of age exceeded 5% and fell below 5%, respectively (4). The timing of each pre-COVID-19 RSV seasons was defined as described in (4). The 2023/24 RSV season covered calendar weeks 47 of 2023 to 10 of 2024, while the 2024/25 RSV season covered weeks 3 to 15 of 2025.

An ecological study was conducted to compare the RSV PR (virological ARI surveillance) and the RSV cumulative incidence (ICOSARI and notification surveillance systems) for infants (< 1 year) and toddles (1 - <5 years) in the 2024/25 RSV season to the previous 2023/24 RSV season and pre-COVID-19 RSV seasons, respectively. RSV cumulative incidence was calculated as the number of RSV cases per 100,000 inhabitants in an RSV season. To quantify changes in RSV cumulative incidence, we calculated risk ratios (RRs) with 95% confidence intervals (CIs). Using ICOSARI data, we further characterised hospitalised RSV cases among infants.

A chi-squared test was used to compare RSV PR (virological ARI surveillance), proportion of RSV diagnoses among SARI cases and proportion of death among hospitalised RSV cases (ICOSARI) in 2024/25 to the previous seasons, and to assess statistical significance of RRs (ICOSARI and notification surveillance systems). Mann–Whitney U test was applied to compare the age of hospitalised RSV cases and the length of hospitalization (ICOSARI). A *p*-value < 0.05 was considered statistically significant. All data analyses were performed using Stata (version 17).

**Virologic SARI Surveillance Study Group investigators**

David Krieger, Jessica Gallisch, Nicolas Schönfeld (Helios Klinikum Emil von Behring GmbH, Klinik für Pneumologie / Lungenklinik Heckeshorn, Berlin Lung Institute, Berlin, Germany); Annette Günther (Helios Klinikum Emil von Behring GmbH, Klinik für Kinder- und Jugendmedizin, Berlin, Germany); Ute Oltmanns (Helios Klinikum Pforzheim GmbH, Klinik für Pneumologie, Germany); Kai Siedler, Marwan Madi (Helios Klinikum Pforzheim, Klinik für Kinder und Jugendliche, Germany); Christoph von Buch (Diakonie Kliniken Bad Kreuznach gGmbH, Pädiatrie, Germany); Mathias Lehmann (Helios Klinikum Krefeld, Klinik für Pneumologie, Schlaf- und Beatmungsmedizin, Germany); Kurt Rasche (Helios Universitätsklinikum Wuppertal, Universität Witten/Herdecke, Klinik für Pneumologie, Allergologie, Schlaf- und Beatmungsmedizin, Germany); Kai O. Hensel (Helios Universitätsklinikum Wuppertal, Universität Witten/Herdecke, Zentrums für Kinder- und Jugendmedizin, Germany); Nikolaus Büchner (Helios Klinikum Duisburg, Klinik für Pneumologie, Schlaf- und Beatmungsmedizin, Germany); Uribe Guzman, Olga Weinberger, Karoline Jungnickel (Helios Klinikum Gifhorn, Pneumologie Medizinische Klinik III, Germany); Benjamin Berlemann, Elke Reutershahn (Helios St. Johannes Klinik Duisburg, Klinik für Kinder und Jugendliche Duisburg, Germany), Wolfram Grüning (Helios Kliniken Schwerin, Klinik für Pneumologie, Germany); Claudius Werner (Helios Kliniken Schwerin, Klinik für Kinder- und Jugendmedizin, Germany); Jens-Uwe Bauer (Helios Klinikum Erfurt, Thoraxzentrum, 1. Medizinische Klinik, Germany); Levente Bejo (Helios Klinikum Hildesheim, Kinderzentrum/Neonatologie, Germany); Tobias Leis (Helios St. Marienberg Klinik Helmstedt, Pneumologie und Beatmungsmedizin, Germany); Selcuk Tasci (Helios Klinikum Bonn/Rhein-Sieg, Klinik für Pneumologie, Schlaf- und Beatmungsmedizin, Germany)

**References**

1. Cai W, Kondgen S, Tolksdorf K, Durrwald R, Schuler E, Biere B, et al. Atypical age distribution and high disease severity in children with RSV infections during two irregular epidemic seasons throughout the COVID-19 pandemic, Germany, 2021 to 2023. Euro Surveill. 2024;29(13).

2. icd.who.int. International statistical classification of diseases and related health problems 10th revision. ICD-10 Online version: 2019 [Internet]. World Health Organization; 2019 [Available from: <https://icd.who.int/browse10/2019/en>.

3. Institute RK. How is the activity of acute respiratory infections monitored in Germany? [Internet]. Robert Koch Institute; [updated 29.09.2025. Available from: <https://www.rki.de/EN/Topics/Infectious-diseases/Acute-respiratory-infections/monitoring.html>.

4. Cai W, Durrwald R, Biere B, Schweiger B, Haas W, Wolff T, et al. Determination of respiratory syncytial virus epidemic seasons by using 95% confidence interval of positivity rates, 2011-2021, Germany. Influenza Other Respir Viruses. 2022;16(5):854–7.
